# ASSESSING MOTOR FUNCTION IN FRAIL OLDER ADULTS IN THEIR HOME SETTING: CHALLENGES AND OPPORTUNITIES

**DOI:** 10.1093/geroni/igad104.1806

**Published:** 2023-12-21

**Authors:** Lijuan Yin, Woojin Song, Jordan Skowronski, Maria Caceres

**Affiliations:** University of Illinois Chicago, Chicago, Illinois, United States; University of Illinois Chicago, Chicago, Illinois, United States; University of Illinois Chicago, Chicago, Illinois, United States; University of Illinois Chicago, Chicago, Illinois, United States

## Abstract

Motor function is essential for older adults to perform daily activities and remain in their home as they age. Mounting evidence suggests that physical activity (PA) helps maintain motor function in older adults. However, few evidence-based PA programs exist for home-bound older adults who have difficulties walking or standing. They are typically excluded from randomized controlled trials (RCT). Evidence is difficult to establish, because of the lack of instruments and protocols for assessing motor function safely and effectively in this population. The goal of this paper is to present challenges and opportunities that we encountered in assessing motor function in 119 participants of the Pro-Home study right before the COVID-19 pandemic. As expected, participants had significant physical limitations. One third of the participants did not feel safe doing balance tests or chair stand test included in the Short Physical Performance Battery. Of those who did the balance tests, two thirds had to use compensatory movement or hold on to an object to maintain balance. Interestingly, 30% of participants had cognitive impairment as defined by 1.5 Standard Deviation below the normative mean measured by NIH Toolbox Cognition Battery, although they passed a six-item cognition screener to be eligible for Pro-Home. The challenges of assessing frail older adults have led to opportunities to adapt assessment protocols. We made instructions clear and easy-to-follow and developed a new measure appropriate and safe for our target, frail older participants. Additional safety measures are needed to facilitate motor function assessments in frail older adults.

